# Design of gradient nanopores in phenolics for ultrafast water permeation†
†Electronic supplementary information (ESI) available: Experimental details and additional characterization (macroscale photographs, SEM images, *etc.*) of phenolic materials prepared under different conditions, and comparison in terms of permeance and BSA and Cyt.c rejections between the phenolic membranes prepared in this work and other membranes. See DOI: 10.1039/c8sc03012j


**DOI:** 10.1039/c8sc03012j

**Published:** 2018-12-11

**Authors:** Leiming Guo, Yazhi Yang, Fang Xu, Qianqian Lan, Mingjie Wei, Yong Wang

**Affiliations:** a State Key Laboratory of Materials-Oriented Chemical Engineering , Jiangsu National Synergetic Innovation Center for Advanced Materials , College of Chemical Engineering , Nanjing Tech University , Nanjing 210009 , Jiangsu , China . Email: yongwang@njtech.edu.cn

## Abstract

Membrane technology is playing a pivotal role in providing potable water to our thirsty planet. However, the strong demand for highly permeable and durable membranes with affordable costs remains. Such membranes are synthesized herein by designing gradient nanopores in low-cost phenolics. The gradient nanopores are achieved by spontaneous assembly of phenolic nanoparticles with gradually enlarged sizes. These particles nucleate and grow as a result of ZnCl_2_-accelerated thermopolymerization of resol in the progressive downward gelating polymer. Subsequent removal of the gelated polymer and ZnCl_2_ exposes the gradient nanopores. The gradient nanopores endow the phenolic structures with unprecedented permselectivity when used in membrane separation, totally rejecting fine particulates down to 5 nm dispersed in water or aggressive solvents while allowing water to permeate up to two orders of magnitude faster than other membranes with similar rejections. Our work opens up an avenue for the rational design and affordable synthesis of ultrafast membranes.

## Abstract 2

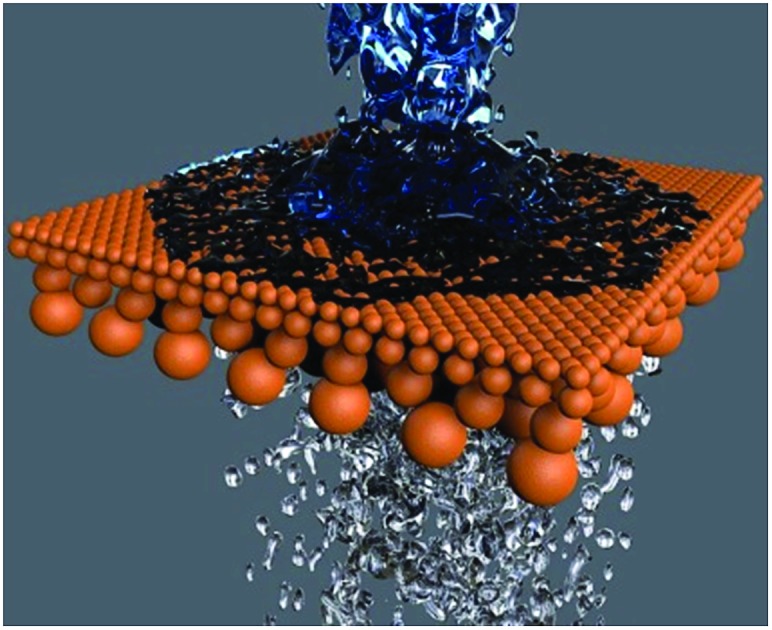
Ultra-permeable and robust membranes are prepared by creating a gradient nanoporous structure in low-cost phenolics, enabled by the spontaneous assembly of gradually enlarged phenolic nanoparticles.

## Introduction

Over 1.8 billion people globally use contaminated sources of drinking water.^1^ As a result, to produce clean and safe water, purification technologies have been extensively used.^2^ As an energy-efficient, easy-to-operate technology with small footprints, membrane separation is ubiquitous nowadays and becoming indispensable in various processes for water purification.^3^ Membranes are designed to discriminate ions, organic molecules (*e.g.*, antibodies), viruses, bacteria, or colloids from water by regulating the pore size and shape and pore wall chemistry, *etc.*^4^ Ideally, membranes are desired to completely reject contaminants while allowing water to pass at lowest resistance, thus further reducing energy consumption and running costs. To this end, tremendous efforts have been made to design new membranes with enhanced water permeabilities with no or little sacrifice of selectivity.^5^–^8^

From the Hagen–Poiseuille equation which correlates the structural parameters of porous membranes with permeability (*P*),^9^

1 *P* = π(*R*^4^Δ*P*)/(8*ηL*)

it can be understood that the thickness (*L*) of the selective layer of the membrane should be minimized to increase the permeability. However, the selective layer also needs to be mechanically robust to withstand the pressure that drives the separation across the membrane. Therefore, asymmetric structures with a thin selective layer on top of a mechanically strong substrate are predominantly applied for membrane separation.^10^–^13^

Thin-film composite reverse osmosis (RO) membranes are best known for their asymmetric structures.^14^ Interfacial polymerization is *in situ* performed on the surface of the polysulfone ultrafiltration (UF) membrane, producing a tight polyamide layer with a thickness of ∼100–400 nm on the UF substrate.^15^,^16^ The thus-produced composite membranes are capable of rejecting ions from water. Recently, the bi-layered composite configuration is receiving new attention. A number of nanomaterials including nanoparticles, nanofibers, and two-dimensional materials, for example, graphene oxides,^17^ MXene,^8^ and C_3_N_4_,^18^ have been assembled on the surface of macroporous substrates to form nanoporous layers with a thickness down to a few tens of nanometers. The composite membranes with the selective layers established from low-dimensional building blocks have shown impressively high water permeability while maintaining good selectivity. Unfortunately, the high costs, cumbersome processing, and problematic stability may significantly limit the scalability and real-world applications of these membranes. Therefore, in the ever-straining situation of global water scarcity, the synthesis of ultra-permeable and robust membranes with tight selectivities from affordable raw materials through simple and efficient processes remains in high demand.

To meet this demand, attention should also be paid to low-cost and easily available materials. Phenolic resins have long been used in many different fields because of their low cost and superior chemical, thermal, and mechanical robustness.^19^ Moreover, a number of nanostructured phenolics, including nanospheres, nanofibers, and mesoporous films, have been synthesized by adopting different precursors and controlling the polymerization process.^20^–^22^ These features, all of which are desired for an ideal membrane, inspire us that phenolics are potential candidates for high-performance membranes. Unfortunately, phenolics are insoluble and infusible and therefore they cannot be processed to membranes *via* conventional methods. Alternatively, in precipitation polymerization, phenolic particles are precipitated from solution and form aggregates with certain porosity depending on the polymerization conditions. We expect that if the precipitation of phenolic particles occurs in a suitably controlled way, there might be a chance to have a well-organized assembly of phenolics with tunable porosity favoring fast mass transfer. Moreover, composite structures with a thin layer on top of a macroporous substrate have been widely used to improve the membrane permeance. And these composite structures frequently suffer from complicated preparation processes and poor interfacial adhesion between the two layers.^23^ Therefore, the demand to develop other membrane configurations, for example, asymmetric membranes with gradient pores remains.^24^ With increasing pore size from the top surface to the bottom, such a structure is expected to exhibit enhanced permeance while maintaining tight rejection.

Based on these considerations, we demonstrate herein the design and synthesis of phenolic-based advanced membranes by establishing a highly gradient nanoporous structure in phenolics. The precipitation polymerization of phenolics is accelerated by ZnCl_2_ and is coupled with the gelation of Pluronic polymer (PEO-*block*-PPO-*block*-PEO block copolymer) with the fast evaporation of the solvent. Under these conditions, phenolic nanoparticles nucleate and grow with gradually increased sizes from the top to the bottom and spontaneously assemble to form membranes with a gradient porous structure. Remarkably, the thus-produced phenolic membranes outperform not only commercial membranes (by a factor of 20–80 in terms of permeance) but also newly developed advanced membranes based on costly 2D materials. Also importantly, the phenolic membranes are selective and robust enough to completely reject fine particulates with a size down to a few nanometers dispersed either in water or in aggressive solvents. The aim of this work is to report an ultrafast membrane for water purification and also to demonstrate the unlimited potentials of low-cost materials in developing ultrafast membranes.

## Results and discussion

### The gradient structure of the phenolic membranes

**Fig. 1:**
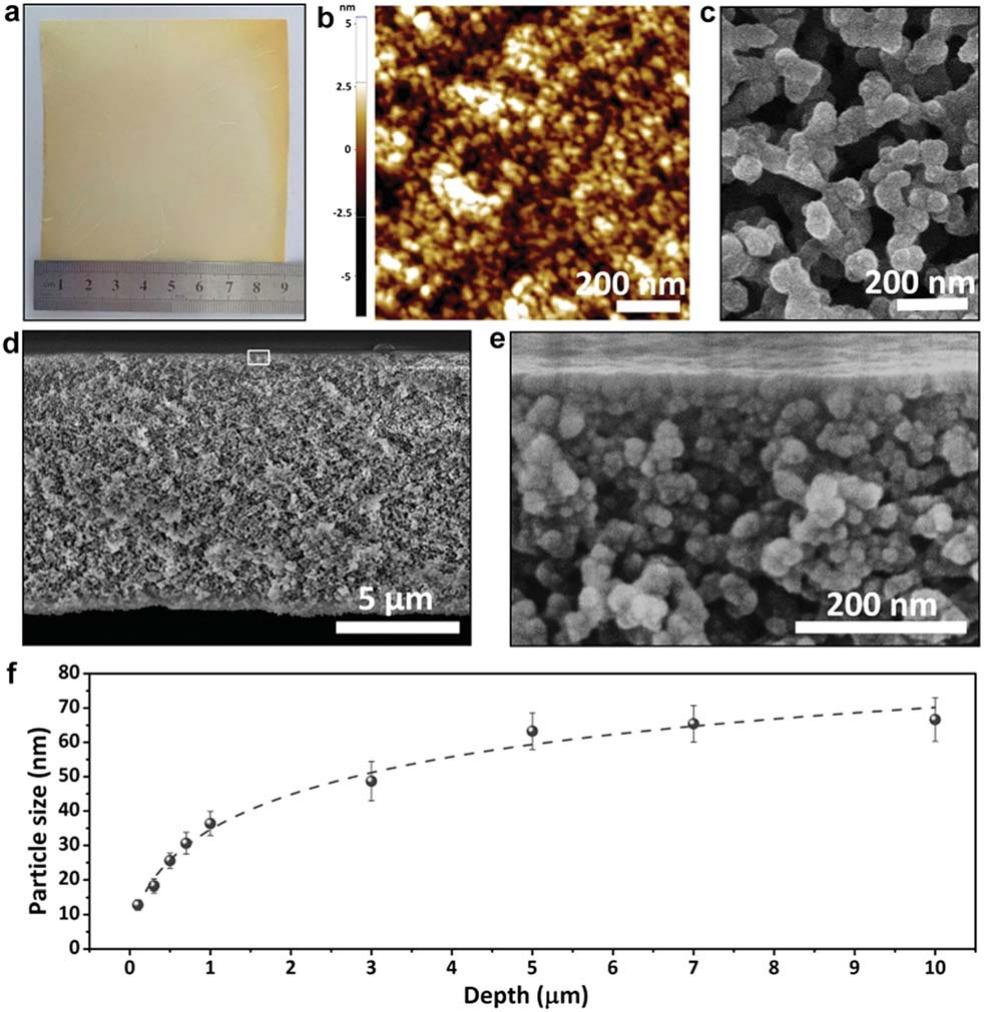
Phenolic membranes with a gradient nanoporous structure. (a) The photograph of the membrane; (b) the AFM topography image of the top surface; (c) the SEM image of the bottom surface; (d) the SEM image of the cross section and (e) the enlarged image of the boxed area in (d); (f) the size of phenolic particles at different depths across the membrane.

To prepare the phenolic membranes, an ethanolic solution containing resol (the precursor of phenolics), Pluronic polymer (P123) and ZnCl_2_ was cast on the surface of a flat substrate and evaporated at 100 °C to produce a thin film supported on the substrate (Fig. S1†). During this heat treatment, resol was thermopolymerized, and the resultant phenolic film containing P123 and ZnCl_2_ exhibited a dense and flat surface (Fig. S2a†). Interestingly, scanning electron microscopy (SEM) revealed that the film was composed of small particles embedded in a dense matrix (Fig. S2b†). We then soaked the phenolic film in H_2_SO_4_ to decompose P123 25 and also to dissolve ZnCl_2_, producing a free-standing, flexible phenolic membrane. Fig. 1a shows the prepared membrane with a lateral size of 9 cm × 9 cm transferred onto a nonwoven polyester support. Atomic force microscopy (AFM) surface topography revealed that the membrane surface was composed of densely packed nanoparticles (Fig. 1b). In contrast, the bottom of the phenolic membrane exhibited a porous structure composed of interconnected spherical particles with a diameter of *ca.* 67 nm (Fig. 1c). The porous structure spanned the entire 10 μm-thick membrane, demonstrating a pronounced gradient porous structure (Fig. 1d). Spherical particles with gradually increased diameters from the top to the bottom were densely assembled (Fig. 1d and e), forming a continuous skeleton of the membrane. Fig. 1f correlates the size of the phenolic particles with their depths in the membrane (the vertical distance to the top surface). The particle size was increased from ∼13 nm at a depth of ∼0.1 μm to ∼26 nm and ∼36 nm at a depth of 0.5 μm and 1 μm, respectively, and was further increased to ∼67 nm at a depth of 10 μm. If the as-synthesized phenolic film was soaked in water instead of H_2_SO_4_, a similar gradient porous structure could also be obtained (Fig. S3†). This method is also able to prepare bulk materials of gradient nanoporous phenolics with a thickness >100 μm simply by increasing the volume of the casting solution. As shown in Fig. S4,† a phenolic membrane with a thickness of ∼150 μm was synthesized. The thick membrane exhibited a very pronounced gradient porous structure as the phenolic particles in the membranes were progressively enlarged from a few tens of nm near the top surface to ∼500 nm on the bottom side.

We tried to directly characterize the pore sizes. However, in the gradient structures, the pores are defined as gaps formed by stacking phenolic particles. These pores are interconnected with each other and do not have a measurable profile. Therefore, the sizes of such highly irregular pores cannot be estimated. Instead, the sizes of spherical phenolic particles from the top to the bottom of the membranes can be relatively easily measured, and the gaps between them are highly related to the particle sizes.

### Interplays among the components in the phenolic films

**Fig. 2:**
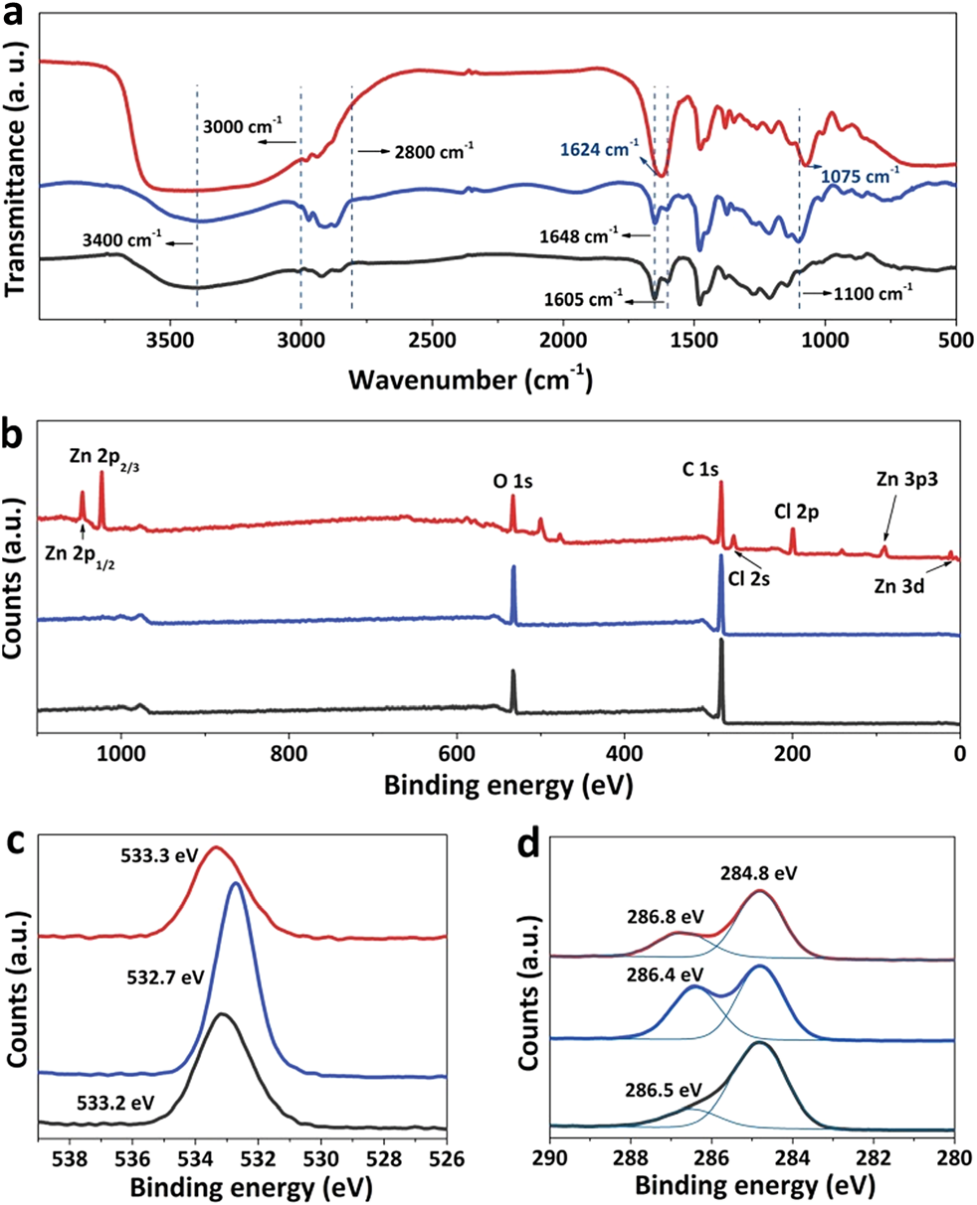
The chemical composition of the phenolic film before (red curve) and after soaking in water (blue curve) or H_2_SO_4_ (black curve). (a) IR spectra, (b) wide-scan XPS spectra, and the corresponding enlarged spectra of (c) O and (d) C.

Infrared (IR) spectroscopy was applied to reveal the change in the chemical composition of the phenolic film before and after soaking in H_2_SO_4_ or water. As shown in Fig. 2a, the broad peaks centered around 3400 cm^–1^ originate from hydroxyl groups,^26^ and the strongest peak of the as-synthesized phenolic film is mainly contributed by water absorbed from the air as a result of the strong hygroscopicity of ZnCl_2_.^27^ A strong peak emerged at 1624 cm^–1^ in the spectrum of the as-synthesized phenolic film, which can be attributed to the interaction between ZnCl_2_ and phenolic group. With the introduction of ZnCl_2_, the aromatic C=C bond in the phenolic film at 1605 cm^–1^ is enhanced due to the facilitated aromatization of the phenolic film by ZnCl_2_ while the deoxygenation effect of ZnCl_2_ weakens the characteristic –C=O bond of the phenolic film at 1648 cm^–1^.^28^ The overlap of the two neighboring peaks leads to a merged peak at 1624 cm^–1^. After the removal of ZnCl_2_ by water or H_2_SO_4_, the merged peak is decomposed to the initial two isolated peaks at 1605 cm^–1^ and 1648 cm^–1^. The peak of C–O stretching of P123 was reported to be located at 1100 cm^–1^.^29^ However, for the as-synthesized phenolic film, this peak is shifted to 1075 cm^–1^ as a result of the complexation of Zn^2+^ to P123.^30^ After complete removal of ZnCl_2_ by water, the peak recovers from 1075 cm^–1^ to 1100 cm^–1^. Moreover, a peak at 1100 cm^–1^ shows up with a weaker intensity for the water-soaked sample, indicating that P123 was only partially removed by water. In contrast, after soaking in H_2_SO_4_, the peak disappears, indicating the complete removal of P123.

X-ray photoelectron spectroscopy (XPS) was further used to analyze the phenolic films before and after soaking. Zinc and chlorine elements are distributed on the surface of the as-synthesized phenolic film but absent in the water- and acid-treated films (Fig. 2b), confirming again that both water and H_2_SO_4_ are able to completely remove ZnCl_2_ from the phenolic films. The peak around 533 eV originates from C–OH of phenolic and ether groups of P123^31^ and exhibits stronger intensity after soaking in water. This is due to the removal of ZnCl_2_, releasing some coordinated –OH groups. The strong peak of this group is also achieved for the H_2_SO_4_-soaked film (Fig. 2c). However, this peak is weaker compared to that of the water-soaked film. Generally, abundant –OH groups are able to be generated in the phenolic film by H_2_SO_4_, which can improve the surface hydrophilicity of the phenolic films. To demonstrate this, water contact angles were further determined to be 83°, 65°, and 41° for the as-synthesized phenolic film and the film after soaking in water and H_2_SO_4_, respectively. It should be noted that the complete decomposition of ether groups of P123 by H_2_SO_4_ leads to a weakened O peak in the spectrum of the acid-treated film although the excessive –OH groups can be produced by H_2_SO_4_. Moreover, the peaks at 284.8 and 286.8 eV are attributed to the carbon atoms of formaldehyde after crosslinking^32^ and ester species (*C–O–C=O) of the phenolic film^33^ in the C 1s spectra, respectively (Fig. 2d). As H_2_SO_4_ is capable of removing the oligomeric phenolic group and further facilitating the crosslinking of phenolics at 100 °C, the H_2_SO_4_-soaked film exhibits the strongest peak around 284.8 eV among all the samples. Meanwhile, the degradation of ester bonds takes place in H_2_SO_4_ at high temperature, resulting in the lowest peak intensity of around 286.5 eV for the H_2_SO_4_-soaked film.

### Mechanism for the development of the gradient structure

**Fig. 3:**
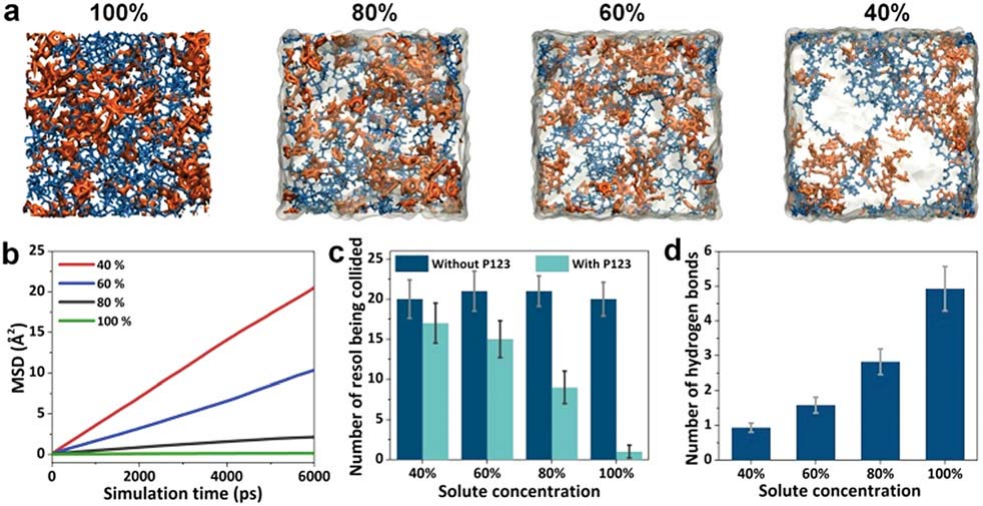
Simulations of the diffusivity and collision of resol molecules. (a) Snapshots of simulation systems with the concentration of solutes decreasing from 100% to 40%. Blue, orange, and transparent grey represent P123, resol, and ethanol, respectively; (b) mean square displacement of the resol molecules; (c) the number of other resol molecules that could be collided by a certain resol molecule; (d) the number of hydrogen bonds between resol molecules and P123.

We carried out molecular dynamics (MD) simulations to investigate the diffusion and reactions of resol molecules under fast evaporation conditions. We changed the concentrations of solutes (P123 and resol with a fixed molar ratio) to describe the progressive evaporation of ethanol. The concentration of solutes was progressively decreased from the top surface to the bottom with the top surface having a 100% concentration. Fig. 3a shows four snapshots from simulations with the concentration of solutes decreasing from 100% to 40%, which depict the state of the compositions at sections with increasing depths in the evaporating solution. We investigated the diffusivity of resol in solution with various concentrations by simulating the change in its mean square displacements (MSDs) with time. As revealed by Fig. 3b, higher concentrations of solutes lead to smaller slopes of the MSD–time plots, indicating lower diffusivity of resol molecules. In the extreme case with a concentration of 100%, resol totally loses its diffusivity, corresponding to the frozen state in which the polymerization of resol is completely terminated. Fig. 3c exhibits the number of other resol molecules being collided with one certain resol molecule within 10 ns, which is an indicator of the rate of resol thermopolymerization leading to the formation of phenolic particles. In the presence of P123, every resol molecule collides with a smaller number of other resol molecules with increasing concentration of solutes. With lower concentrations, because of the higher diffusivity as discussed above, resol molecules can penetrate through the surrounding P123 chains and collide with more other resol molecules, producing larger phenolic particles. In contrast, higher solute concentrations lead to smaller phenolic particles. That is to say, phenolic particles are progressively enlarged with increasing depths in the film, consistent with our experimental observations. Moreover, simulations of the collision of resol molecules in the absence of P123 show that the numbers of collided resol molecules remain almost constant under different concentrations, clearly indicating the strong suppression of the diffusion of resol into P123. In addition, the numbers of hydrogen bonds between P123 and resol increase with the concentration of solutes (Fig. 3d). This means that with the evaporation of ethanol, the interaction between P123 and resol is enhanced, thus weakening the diffusion of resol and consequently the growth of phenolic particles. Note that, for simplicity, ZnCl_2_ was not included in the simulation systems, and this simplification does not influence the reliability of the simulated results because ZnCl_2_, as a small molecule, would not evidently change the relative diffusivity and collision of solute molecules.

**Fig. 4:**
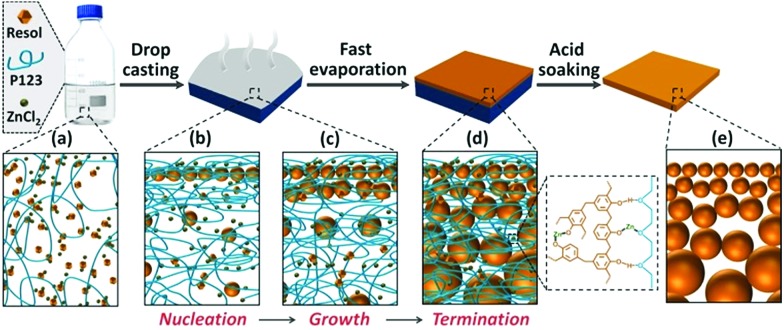
Illustration of the formation of the gradient nanoporous phenolics. (a) Casting the ethanolic solution of resol, P123 and ZnCl_2_ onto the substrate; (b and c) heating initiated the thermopolymerization of resol and gelation of P123 with the fast evaporation of ethanol, leading to the nucleation (b) and growth (c) of phenolic nanoparticles; (d) the phenolic particles were frozen in the gelated P123 with the complete evaporation of ethanol and the growth was terminated; (e) the gradient nanoporous structure was produced by removing P123 and ZnCl_2_ with H_2_SO_4_.

Simulations reveal the influence of the solute concentrations on the diffusivity and collision of resol molecules, which helps to elucidate the mechanism for the formation of the gradient structure. At a temperature of 100 °C, thermopolymerization of resol in the solution takes place, producing phenolic polymers with a crosslinking structure.^28^ Phenolics are not soluble in ethanol and tend to nucleate in solution as primary particles (Fig. 4a and b). At such a high temperature, ethanol evaporates very fast, and the evaporation starts from the surface of the solution. This leads to the gelation of P123, which also starts from the solution surface and propagates to the interior of the solution. The phenolic particles are, therefore, “frozen” in the gelated surface and no longer grow because unreacted resol molecules are also trapped and cannot diffuse to come into contact with the phenolic nucleates within a reasonable timeframe (which is actually due to the interruption of the supply of resol). However, ethanol in the interior of the solution takes longer time to evaporate and the diffusion of resol there is less suppressed, allowing phenolic nucleates to grow with the continuous supply of resol until being frozen by gelated P123 (Fig. 4c and d). Therefore, phenolic particles with increasing sizes from the top surface to the bottom side are formed in the finally obtained structure, producing a composite film with a superstructure of aggregated phenolic particles embedded in P123 (Fig. S2b†). In the subsequent soaking in H_2_SO_4_, both P123 and ZnCl_2_ are removed, leaving behind the network composed of interconnected phenolic particles as a nanoporous membrane with gaps among the particles as the pores tapered from the bottom side to the top side (Fig. 4e).

### Separation performance of the phenolic membranes

**Fig. 5:**
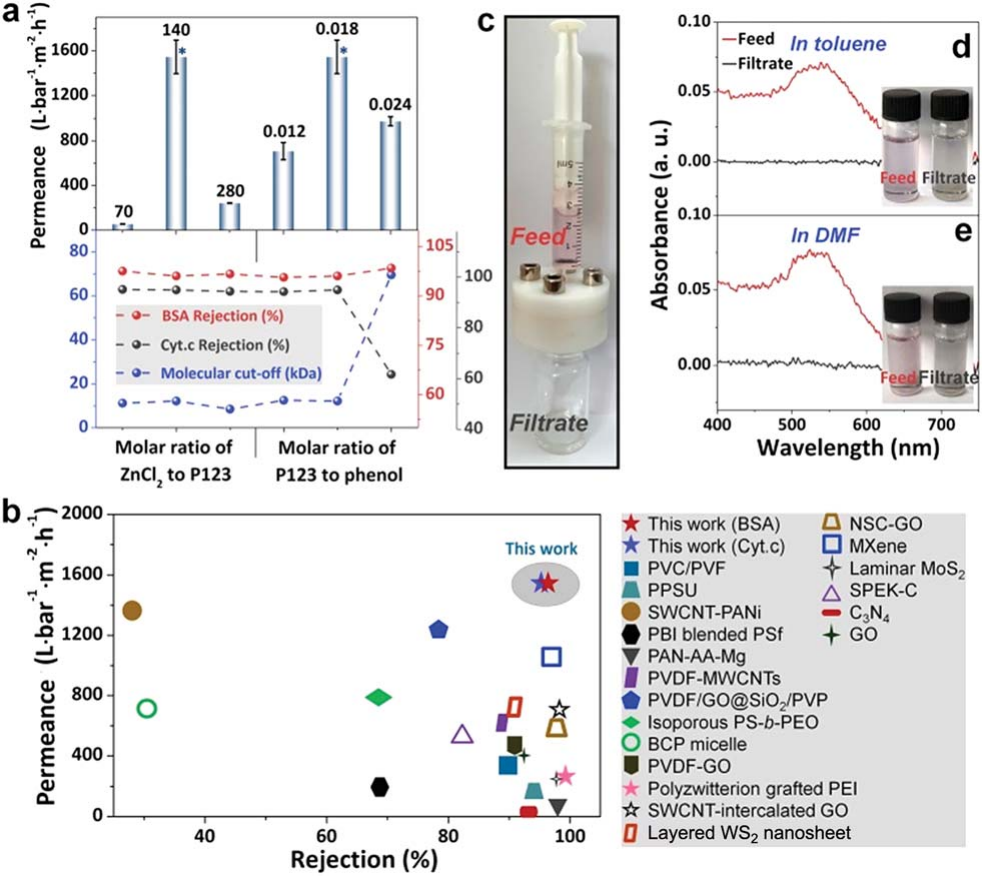
Separation performance of the phenolic membranes. (a) The permeance, rejections to BSA and Cyt.c and MWCOs of the membranes prepared with different compositions in the resol solution. The performances marked with asterisks are acquired from the same membranes; (b) comparison of the phenolic membrane prepared under the optimized conditions with other membranes in terms of rejection and water permeance; (c) the filtration module for separating 5 nm gold nanoparticles in organic solvents; (d, e) the UV-vis spectra of the feeds and filtrates involved in the separation of 5 nm gold nanoparticles in (d) toluene and (e) DMF.

The produced phenolics feature a gradient nanoporous structure, which is highly desired in membrane separation because it promises fast permeation at little cost of separation selectivity. The phenolic membrane with a thickness of ∼10 μm was robust enough to be used in pressure-driven filtration. The water permeance of the water-soaked membrane was as small as 1.9 L bar^–1^ m^–2^ h^–1^. This should be ascribed to the incomplete removal of P123 from the gaps among the phenolic particles, especially in the top layer. In contrast, the membrane prepared by soaking in H_2_SO_4_ exhibited a permeance of ∼1547 L bar^–1^ m^–2^ h^–1^, and its rejections to bovine serum albumin (BSA, *M*_w_ = 66 kDa) and cytochrome C (Cyt.c, *M*_w_ = 12.4 kDa) were measured to be 96% and 95%, respectively (Fig. 5a). As the size of BSA and Cyt.c is 14 nm × 3.8 nm × 3.8 nm 34 and 3.8 nm × 2.5 nm × 2.5 nm,^6^ respectively, the effective diameter of the pores on the top surface of this phenolic membrane can be estimated to be <4 nm. Furthermore, the molecular weight cut-off (MWCO) of the membrane, which is defined as the equivalent molecular weight of the smallest solute that would exhibit 90% rejection, was determined to be 12.2k Da.

Like other polymeric membranes, there is also a flux decline for the gradient phenolic membranes due to structure compression during filtration. However, as shown in Fig. S5,† a steady permeance can be achieved within 15 min of operation. Moreover, the steady permeance accounts for more than 80% of the initial value. Therefore, the gradient phenolic membranes are relatively stable at the operation pressure investigated here.

We compared the phenolic membranes with gradient porosities prepared in this work with other membranes prepared from different materials ranging from conventional polymers to newly emerged 2D materials. Strikingly, although the phenolic membranes were prepared from cheap materials through a relatively convenient process, they exhibit ∼20 to 80 times higher water permeabilities than typical commercial UF membranes with a nominated MWCO of 10 or 20k Da (water permeance ∼20–70 L bar^–1^ m^–2^ h^–1^).^35^ Moreover, compared to newly developed membranes based on costly 2D building blocks, for example, graphene oxide, WS_2_, and MXene, our phenolic membranes with gradient porosities also show ∼1.5–6 times higher permeabilities and similar rejections (Fig. 5b and Table S1†).

Phenolics possess a crosslinked structure with outstanding thermal and chemical stability, implying that the phenolic membranes have great potential to be used under harsh conditions, for example, in aggressive organic solvents. The phenolic membrane was used to filtrate 5 nm gold nanoparticles dispersed in toluene and dimethyl formamide (DMF) (Fig. 5c). The gold particles dispersed either in toluene or in DMF displayed a pink color and exhibited an absorption peak around 539 nm. After filtration, the collected permeates were colorless in both cases and there were no noticeable absorption peaks in the UV-vis spectra (Fig. 5d and e). It should be noted that no damage occurred to the membranes during the course of filtration, and no adsorption of gold particles on the membrane was observed. Therefore, we conclude that the phenolic membranes are robust enough to tolerate both nonpolar and polar organic solvents and are able to reject fine particles as small as 5 nm.

### Tunability of the gradient structures and the separation performances

Fast evaporation of the solvent during the thermopolymerization of resol is essential for the formation of increasingly enlarged phenolic particles. The growth of phenolic nucleates is dependent on the availability of resol molecules which is determined by the gelating state of P123. Moreover, the gelation of P123 is caused by the evaporation of the solvent. If the resol solution was controlled to be slowly evaporated at 20 °C and then subjected to thermopolymerization at 100 °C, in which the gelation of P123 was finished before thermopolymerization, the structure obtained after H_2_SO_4_ soaking was a homogenous film composed of small phenolic particles without a noticeable gradient porous structure (Fig. S6†). In the gelated P123 matrix, resol molecules are trapped and molecular diffusion can only occur within a very local and limited range even at a temperature of 100 °C. As a result, adjacent resol molecules are locally polymerized, forming small phenolic particles which do not grow because of the termination of the supply of resol.

Both ZnCl_2_ and P123 play critical roles in the synthesis of the gradient nanoporous phenolics. ZnCl_2_ stabilizes the two polymers, the phenolic film and P123, to prevent uncontrolled macrophase separation and also accelerates the thermopolymerization process to enable fast nucleation of phenolics before the termination of resol supply by the gelating P123. P123 provides a gelating environment to control the growth of the phenolic nucleates. Syntheses without either ZnCl_2_ or P123 produced dense films after H_2_SO_4_ soaking and no pores could be observed under SEM (Fig. S7 and S8†), confirming the importance of both ZnCl_2_ and P123 in the creation of the gradient porous structure.

**Fig. 6:**
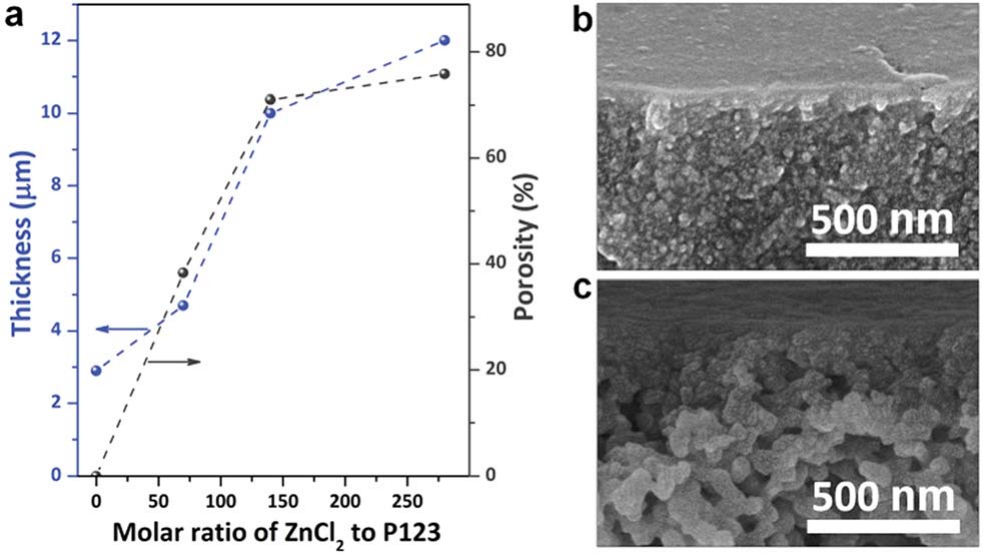
Phenolic membranes prepared at different molar ratios of ZnCl_2_ to P123. (a) Thickness and porosity; (b, c) the cross-sectional SEM images detailing the morphology near the top surface of the membrane prepared at the molar ratios of (b) 70 and (c) 280.

We found that ZnCl_2_ significantly accelerated the thermopolymerization of resol, which should be ascribed to the strong dehydration effect of ZnCl_2_.^33^ When thermopolymerization was performed in the absence of ZnCl_2_, the resol solution took more than 2 h to be completely solidified. However, in the presence of ZnCl_2_ with a molar ratio of ZnCl_2_ to P123 of 140 the resol solution was completely solidified within 10 min. The faster polymerization in the presence of ZnCl_2_ is vividly confirmed by the darker color of the phenolic films compared to the one prepared without ZnCl_2_ as a darker color implies a higher degree of polymerization. Moreover, higher dosages of ZnCl_2_ accelerate the polymerization to greater degrees (Fig. S9†). In addition, ZnCl_2_ dosages strongly influence the porosity of the resulted phenolic membranes. The phenolic structure prepared in the absence of ZnCl_2_ is nonporous and exhibits a thickness of 2.9 μm. As shown in Fig. 6a, there is an evident increase in the thickness of phenolic membranes with increasing ZnCl_2_ dosages (described as molar ratios of ZnCl_2_ to P123). For instance, the thickness is increased to 4.7 and 12.0 μm at a molar ratio of 70 and 280, respectively. Considering that the increase in thickness is caused by the formation of pores in the phenolic structure with the introduction of ZnCl_2_, we can easily estimate the porosity of the phenolic membranes by comparing their thicknesses with that of the phenolic structure produced in the absence of ZnCl_2_.^36^ The porosity of the phenolic membranes prepared at a molar ratio of 70, 140, and 280 was thus estimated to be 38.3, 71.0, and 75.8% (ESI S10 and Fig. S11†), respectively, indicating that higher ZnCl_2_ dosages lead to larger porosities. Cross-sectional SEM examinations confirm that the phenolic membrane prepared at the molar ratio of 70 exhibits a relatively dense morphology while the membrane prepared at the molar ratio of 280 is highly porous (Fig. 6b and c). At low ZnCl_2_ dosages, *e.g.*, at the molar ratio of 70, the concentration of ZnCl_2_ was low, and thermopolymerization slowly took place. Therefore, phenolic nucleates were trapped in the gelated P123 before adequate growth with the evaporation of ethanol, producing much denser phenolic films. However, at the molar ratio of 280, the high dosage of ZnCl_2_ greatly accelerated the polymerization of resol, and phenolic particles nucleated earlier and grew faster, thus producing a highly gradient structure.

The dosages of ZnCl_2_ and P123 influence the morphology and consequently the water permeability of the resultant phenolic membranes. As shown in Fig. 6a, when the molar ratio of ZnCl_2_ to P123 was changed from 140 to 70 and to 280, the permeance was changed to 55 and 242 L bar^–1^ m^–2^ h^–1^, respectively. As discussed above, the molar ratio of 70 produced a much denser phenolic membrane without evident gradient pores (Fig. 6b), giving rise to very low water permeance. In contrast, at the molar ratio of 280, although the membrane exhibited a pronounced gradient structure, the quick nucleation and growth of the phenolics would produce a top layer of densely packed smaller phenolic particles (Fig. 6c), also leading to decreased permeance.

We further investigated the impact of molar ratio of P123 to resol on the membrane performances. Since resol has a wide distribution of molecular weight, phenol is generally replaced by resol to calculate the molar ratio of P123 to resol (see Experiment details).^37^ When the molar ratio of P123 to phenol was 0.012, the membrane retained an apparent gradient porous structure but displayed a reduced permeance of 707 L bar^–1^ m^–2^ h^–1^ (Fig. S12a and b†). At lower concentration of P123, gelation took place at a lower rate, allowing phenolic particles to grow for longer duration and become larger. However, the reduced concentration of P123 also weakened the stabilization of phenolic nucleates, which had a stronger tendency to aggregate with adjacent ones before adequate growth. Consequently, a decreased permeance was obtained. Anyway, the thus-produced membrane exhibited rejections to BSA and Cyt.c as well as MWCOs similar to those of the membrane prepared at a molar ratio of 0.018, which is attributed to the similar pore size in the top layer of the membrane. However, when the molar ratio of P123 to phenol was increased to 0.024, the porous structure was much less pronounced as revealed by the cross-sectional SEM images (Fig. S12c and d†). With a higher P123 concentration, the gelation of P123 occurred faster, and the growth of phenolic particles in the bulk solution was terminated earlier, thus forming a less pronounced gradient porous structure and consequently a thinner membrane (∼6.7 μm in thickness). In addition, the presence of more P123 in the as-synthesized phenolic film led to a looser assembly of phenolic nanoparticles, and consequently, the gaps between the nanoparticles, that is, the pores in the phenolic membrane obtained after soaking in H_2_SO_4_, were somewhat enlarged. Consequently, the membrane exhibited reduced water permeance and also decreased rejection. It should be noted that only in a relatively narrow window of the synthetic conditions can we obtain robust phenolic membranes giving reproducibly high permeance. For example, too much P123 caused defects in membranes, while less P123 could not provide a gelation environment for the formation of gradient phenolics. Similarly, if the molar ratio of ZnCl_2_ to P123 was less than 70, the produced phenolic membranes became hard while too much ZnCl_2_ made the membrane fragile, and both are adverse for reliable membrane separation.

## Conclusions

In summary, gradient nanopores are produced by controlling the thermopolymerization of resol in the mixture of Pluronic polymer (P123) and ZnCl_2_. With the fast evaporation of the solvent, the ZnCl_2_-accelerated thermopolymerization of resol induces the nucleation of phenolics as nanoparticles. They continue to grow until the gelating P123 terminates the supply of resol, thus forming phenolic nanoparticles with increasing sizes from the top to the bottom. These phenolic particles further assemble to form a network with the stabilization effect of P123 and ZnCl_2_. After removal of P123 and ZnCl_2_, phenolics with a gradient nanoporous structure are formed. The thus-produced phenolic structures exhibit unprecedented separation performances when used as membranes. They show ∼20–80 times higher water permeance than commercial membranes with similar rejections. Impressively, they also greatly outperform membranes based on expensive 2D materials by a factor of ∼1.5–6 in terms of water permeance while their selectivities being comparable. Furthermore, the phenolic membranes are robust enough to operate in aggressive organic solvents. Such a gradient nanoporous structure combined with excellent chemical and thermal stability makes the thus-produced phenolic superstructures an exciting candidate for applications in other areas including batteries, supercapacitors, catalysis, *etc.*

## Conflicts of interest

There are no conflicts to declare.

## Supplementary Material

Supplementary information

## References

[cit1] http://www.un.org/sustainabledevelopment/water-and-sanitation/ .

[cit2] Werber J. R., Osuji C. O., Elimelech M. (2016). Nat. Rev. Mater..

[cit3] Ling S., Qin Z., Huang W., Cao S., Kaplan D. L., Buehler M. J. (2017). Sci. Adv..

[cit4] Lee A., Elam J. W., Darling S. B. (2016). Environ. Sci.: Water Res. Technol..

[cit5] Peinemann K.-V., Abetza V., Simon P. F. (2007). Nat. Mater..

[cit6] Peng X., Jin J., Nakamura Y., Ohno T., Ichinose I. (2009). Nat. Nanotechnol..

[cit7] Kandambeth S., Biswal B. P., Chaudhari H. D., Rout K. C., Kunjattu H. S., Mitra S., Karak S., Das A., Mukherjee R., Kharul U. K., Banerjee R. (2017). Adv. Mater..

[cit8] Ding L., Wei Y., Wang Y., Chen H., Caro J., Wang H. (2017). Angew. Chem., Int. Ed..

[cit9] Robinson J. P., Tarleton E. S., Millington C. R., Nijmeijer A. (2004). J. Membr. Sci..

[cit10] Phillip W. A., O'Neill B., Rodwogin M., Hillmyer M. A., Cussler E. L. (2010). ACS Appl. Mater. Interfaces.

[cit11] Querelle S. E., Jackson E. A., Cussler E. L., Hillmyer M. A. (2013). ACS Appl. Mater. Interfaces.

[cit12] Sankhala K., Koll J., Radjabian M., Handge U. A., Abetz V. (2017). Adv. Mater. Interfaces.

[cit13] Guo L., Wang Y. (2014). Chem. Commun..

[cit14] Fritzmann C., Löwenberg J., Wintgens T., Melin T. (2007). Desalination.

[cit15] Ghosh A. K., Hoek E. M. V. (2009). J. Membr. Sci..

[cit16] Xu G.-R., Wang J.-N., Li C.-J. (2013). Desalination.

[cit17] Li H., Song Z., Zhang X., Huang Y., Li S., Mao Y., Ploehn H. J., Bao Y., Mao Y. (2013). Science.

[cit18] Wang Y., Li L., Wei Y., Xue J., Chen H., Ding L., Caro J., Wang H. (2017). Angew. Chem., Int. Ed..

[cit19] Hirano K., Asami M. (2013). React. Funct. Polym..

[cit20] Sun H., Wang Q., Geng H., Li B., Li Y., Wu Q.-H., Fan J., Yang Y. (2016). Electrochim. Acta.

[cit21] Liu J., Qiao S., Liu H., Chen J., Orpe A., Zhao D., Lu G. (2011). Angew. Chem., Int. Ed..

[cit22] Zhang J., Qiao Z., Mahurin S., Jiang X., Chai S., Lu H., Nelson K., Dai S. (2015). Angew. Chem., Int. Ed..

[cit23] Lau W. J., Ismail A. F., Misdan N., Kassim M. A. (2012). Desalination.

[cit24] Ulbricht M. (2006). Polymer.

[cit25] Yang C.-M., Zibrowius B., Schmidt W., Schüth F. (2003). Chem. Mater..

[cit26] Meng Y., Gu D., Zhang F., Shi Y., Cheng L., Feng D., Wu Z., Chen Z., Wan Y., Stein A., Zhao D. (2006). Chem. Mater..

[cit27] Ji L., Medford A. J., Zhang X. (2009). Polymer.

[cit28] Yu Z.-L., Li G.-C., Fechler N., Yang N., Ma Z.-Y., Wang X., Antonietti M., Yu S.-H. (2016). Angew. Chem., Int. Ed..

[cit29] Zhuang X., Qian X., Lv J., Wan Y. (2010). Appl. Surf. Sci..

[cit30] Wang Z., Wang M., Wu G., Wu D., Wu A. (2014). Dalton Trans..

[cit31] Goworek J., Swiątkowski A., Biniak S. (1997). Langmuir.

[cit32] Kimura T., Emre A. M., Kato K., Hayashi Y. (2013). J. Mater. Chem. A.

[cit33] Nabae Y., Sonoda M., Yamauchi C., Hosaka Y., Isoda A., Aoki T. (2014). Catal. Sci. Technol..

[cit34] Qiu X., Yu H., Karunakaran M., Pradeep N., Nunes S. P., Peinemann K.-V. (2013). ACS Nano.

[cit35] Koch ultrafiltration membranes. http://www.kochmembrane.com/Membrane-Products/Hollow-Fiber/Ultrafiltration.aspx.

[cit36] Yan N., Wang Y. (2015). Soft Matter.

[cit37] Meng Y., Gu D., Zhang F., Shi Y., Yang H., Li Z., Yu C., Tu B., Zhao D. (2005). Angew. Chem., Int. Ed..

