# Supplementary material for: Design of gradient nanopores in phenolics for ultrafast water permeation
Source: Chem Sci. 2018 Dec 11;10(7):2093–100. doi: 10.1039/c8sc03012j (PMC6375355; doi:10.1039/c8sc03012j)
Supplement: Supplementary file 1 [file SC-010-C8SC03012J-s001.pdf]

Electronic Supplementary Material (ESI) for Chemical Science.
This journal is © The Royal Society of Chemistry 2018

Supplementary Information

for

Design of gradient nanopores in phenolics for ultrafast water permeation

Leiming Guo,[§] Yazhi Yang, Fang Xu, Qianqian Lan, Mingjie Wei,

and Yong Wang[✉]

State Key Laboratory of Materials-Oriented Chemical Engineering, Jiangsu National Synergetic Innovation Center for Advanced Materials, and College of Chemical Engineering, Nanjing Tech University, 210009 Nanjing, Jiangsu, China

[§] Present address: Institut für Chemie neuer Materialien, Universität Osnabrück, Barbarastr. 7, 49069 Osnabrück, Germany

[✉]Corresponding author

E-mail: yongwang@njtech.edu.cn

Tel: 0086-25-8317 2247

Fax: 0086-25-8317 2292

Experimental details

Materials. Phenol (≥ 99%), aqueous formaldehyde solutions (37 wt%), $ZnCl_2$ (≥ 98%), NaOH (≥ 96%), hydrochloric acid (36-37 wt%), ethanol (≥ 99.8%), purified water (conductivity: 8-20 μs/cm), and 98 wt% H_2SO_4 were all purchased from local suppliers. PEO-*block*-PPO-*block*-PEO triblock copolymer (M_w = 5800 g/mol and the degree of polymerization for each PEO block and PPO block is 20 and 70, respectively, commercially known as P123) was obtained from Sigma-Aldrich. Bovine serum albumin (BSA, molecular weight of 67 kDa, purity > 97%), cytochrome c (Cyt.c, molecular weight of 12.4 kDa, purity > 95%), and dextrans with four different molecular weights (10 kDa, 40 kDa, 70 kDa, and 500 kDa) were obtained from Sigma-Aldrich. Monodispersed gold nanoparticles with a diameter of 5 nm dispersed in toluene and dimethyl formamide (DMF) were purchased from Nanjing Nanoeast Biotech Co. Ltd. All chemicals were used without further purification.

Preparation of resol solutions. Resol was synthesized from phenol and formaldehyde by a base-catalyzed polymerization method, as described elsewhere.[1] In a typical synthesis process, 0.610 g (6.5 mmol) phenol was melted at 45 ºC at first, and then 0.130 g of 20 wt% aqueous NaOH was added by stirring for 10 min. Subsequently, 1.054 g of 37 wt% formaldehyde was added to the above solutions dropwise. After stirring at 75 ºC for 1 h, the reaction mixture was further cooled to the rt followed by the pH adjustment with 0.6 M HCl toward neutral (pH=7). The mixture was dried under vacuum at

45 ºC for 24 h to remove water. The as-prepared resol was redissolved with 5 g ethanol, which was then filtered with 0.22 μm syringe filters to exclude out the insoluble NaCl precipitates from ethanolic solutions. After then, 0.678 g P123 was dissolved in 10 g ethanol, which was further transferred into ethanolic resol solutions to produce the ethanolic mixture containing resol and P123. The molar ratio of P123 to phenol was 0.018. Before use, such solutions, roughly having a total concentration of resol and P123 of 10 wt%, were stirred for at least 12 h to yield homogeneous solutions. However, when the molar ratio of P123 to phenol was required to be 0.012 and 0.024, the dosages of P123 were 0.452 and 0.904 g, respectively. In the control experiment, P123 was replaced with the same amount of PEO homopolymer (0.678 g) in the resol solution, and all the other experimental conditions including thermopolymerization and acid soaking remained unchanged except that PEO was dissolved in ethanol at 60 ºC to ensure a homogenous resol/PEO in ethanol solution.

Fabrication of phenolic membranes. To fabricate the phenolic membranes, the ethanolic solutions containing resol and P123 were diluted with ethanol to have a concentration of 5% (Specifically, to prepare 150 μm-thick phenolic membranes, the solution with the 10 % concentration was used). To 1 g of the solutions, different dosages (0.034, 0.068 and 0.135 g) of $ZnCl_2$ were added, and the molar ratios of $ZnCl_2$ to P123 in these solutions were determined to be 70, 140 and 280, respectively. Typically, the ethanolic solutions with the

molar ratio of P123 to phenol and the molar ratio of $ZnCl_2$ to P123 of 0.018 and 140, respectively, were used in this work if not otherwise stated. 100 μL solutions were drop-cast on the cleaned Si or glass substrates at *rt* and then instantly transferred on a hot plate into an oven preheated to 100 ºC for the thermopolymerization of resol. After 12 h, the substrates together with the formed structures were soaked in 55 wt% H_2SO_4 at 100 ºC for 2 h or in water at rt for 2 h to remove P123 and $ZnCl_2$. In a control experiment with slow evaporation of the solvent, the resol solution containing P123 and $ZnCl_2$ cast on the silicon substrate was placed in a dry cabinet with a relative humidity of ~ 10% at the temperature of 20 ºC, and was covered with a Petri dish to slowly evaporate ethanol for two days. The sample was further dried in vacuum at 25 ºC for 2 h, and was then subjected to thermopolymerization and acid soaking as described above.

Simulations. All the MD simulations were carried out using the large-scale atomic/molecular massively parallel simulator (LAMMPS) package.[2] The polymer consistent force field (PCFF)[3-5] was adopted in all simulations, which is suitable for small organic molecules and polymers. The total potential energy of a system can be defined as Eq. (S1).

$$E_{\text{total}}=E_{\text{valence}}+E_{\text{cross-term}}+E_{\text{non-bond}} \tag{S1}$$

The E_{valence} comprises bond stretching, angle, torsion, and out-of-plane energies. The $E_{\text{cross-term}}$ presents bond length and angle changes. For non-bond energies ($E_{\text{non-bond}}$), they are divided into LJ (9-6) van der Waals(*vdW*)

and Coulombic interactions. The long-range electrostatic interactions are computed by using the particle-particle particle-mesh (PPPM) algorithm with a root mean square accuracy of 10^{-4}. The cutoffs for *vdW* and Coulombic interaction are set as 12 Å. The periodic boundary conditions (PBC) were applied in all three directions for all simulation cubic boxes. Initially, for each simulation, the energy of system was minimized for 1000 steps. Then a pressure of 1 atm was applied to all dimensions to ensure that each system reaches its equilibrium density. After that, the simulations were performed under the NVT canonical ensemble for 13 ns. The first 3 ns was used to equilibrium and the last 10 ns was conducted for data collection. The thermostat was set to 373.15 K during the entire simulations, which is identical to the thermopolymerization temperature in the experiment. We constructed four systems containing resol, P123, and ethanol with four concentrations of the solute (P123+resol). Each system contains 34 resol molecules and two P123 chains, corresponding to the molar ratio of P123 to phenol of 0.018 used in the experiment. The numbers of ethanol molecules were added to 941, 418, 157 and 0, respectively, according to the different solute concentrations of 40%, 60%, 80% and 100%. The different concentrations represent the concentration gradient from the top surface of the solution to the bottom with the top surface having a 100% concentration.

Characterizations. A field-emission scanning electron microscope (SEM, Hitachi S4800) was used to probe the surfaces and the cross sections of the samples at an accelerating voltage of 5 kV. Before SEM examinations the samples were sputter-coated with a thin layer Pd/Pt alloy to enhance the

conductivity. We took SEM images at a series of depths (0.1-10 μm) on the cross section of the 10 μm-thick membrane. From the SEM image of each depth, we measured at least 50 particles to determine the average sizes of phenolic particles, thus correlating the particle sizes with depths across the phenolic membrane. For the transmission electron microscopy (TEM) observations, the membrane samples were ground into powders which were dispersed in ethanol followed by sonication under the power of 100 W for 30 min. A droplet of the suspension was dropped onto TEM grids. TEM observations were carried out on a JEM-2100 microscope operated at 200 kV. The surface topography of the samples was examined by atomic force microscopy (XE-100, Park Systems). Fourier transformation infrared (FTIR) spectra were obtained from a Nicolet 8700 infrared spectrometer in the attenuated total reflection (ATR) mode. X-ray photoelectron spectroscopy (XPS) measurements were carried out on an ESCALAB 250 XPS system (Thermo Scientic) using a monochromatic Al Kα X-ray source with the reference of C 1s line of aliphatic carbon, set at 284.8 eV. The surface wettability of the samples was characterized by testing their water contact angles (WCAs). WCAs were obtained from a contact angle goniometer (Dropmeter A-100, Maist). For every sample, the WCAs of at least 3 positions were tested and the average WCAs were reported. The N_2 adsorption-desorption isotherm was obtained from a surface area and porosity analyzer (Micromeritics, ASAP-2020) at 77 K. Before measurements, the phenolic

membrane was degassed in vacuum at 120 °C for 6 h. Specific surface areas (S_{BET}) are derived from the Brunauer−Emmett-Teller (BET) method using the adsorption data at p/p_0 = 0-1.0. The Pore size distribution is derived from the adsorption branch by using the Barrett−Joyner−Halenda (BJH) model.

Filtration and separation tests. Permeance and rejection tests were performed on a Millipore filtration cell (Amicon 8003, Millipore Co.) at a stirring speed of 600 rpm and a pressure of 0.4 bar. The filtration cell has a working volume of 3 mL and an effective membrane area of 0.9 cm^2. The phenolic membranes were attached on the support of polyester non-woven before fitting into the filtration cell. A pre-compaction at 0.4 bar was carried out to obtain a stable water flux, and then the permeance of the membrane was recorded. BSA and Cyt. C were dissolved in phosphate buffer (pH=7.4) at concentrations of 0.5 g/L and 0.02 g/L, respectively, and were used to probe the retentions of membranes prepared at different conditions. The BSA and Cyt.c concentrations in feeds and filtrates were monitored using a UV-vis absorption spectrometer (NanoDrop 2000c, Thermo) and the intensities of the BSA and Cyt.c characteristic peaks at the wavelength of 280 and 405 nm of the feeds and filtrates were compared to determine the retention rates of BSA and Cyt.c of each membrane.

Four dextrans with molecular weights of 10 kDa, 40 kDa, 70 kDa, and 500 kDa were mixed in water at a concentration of 2.5, 1.0, 1.0 and 2.0 g/L, respectively, and used to determine the molecular weight cut-off (MWCO) of

the membranes. The concentrations of dextrans with different molecular weights were analyzed by gel permeation chromatography (GPC, Waters 1515).

To investigate the separation performances of the phenolic membranes in organic solvent, the membranes were used to filtration solutions of 5-nm gold nanoparticles dispersed in toluene and DMF were used. The feeds and filtrates were monitored using the UV-vis absorption spectrometer and the intensities of the characteristic peaks at the wavelength of around 539 nm of the feeds and filtrates were compared to determine the rejection rates of gold nanoparticles of different membranes.

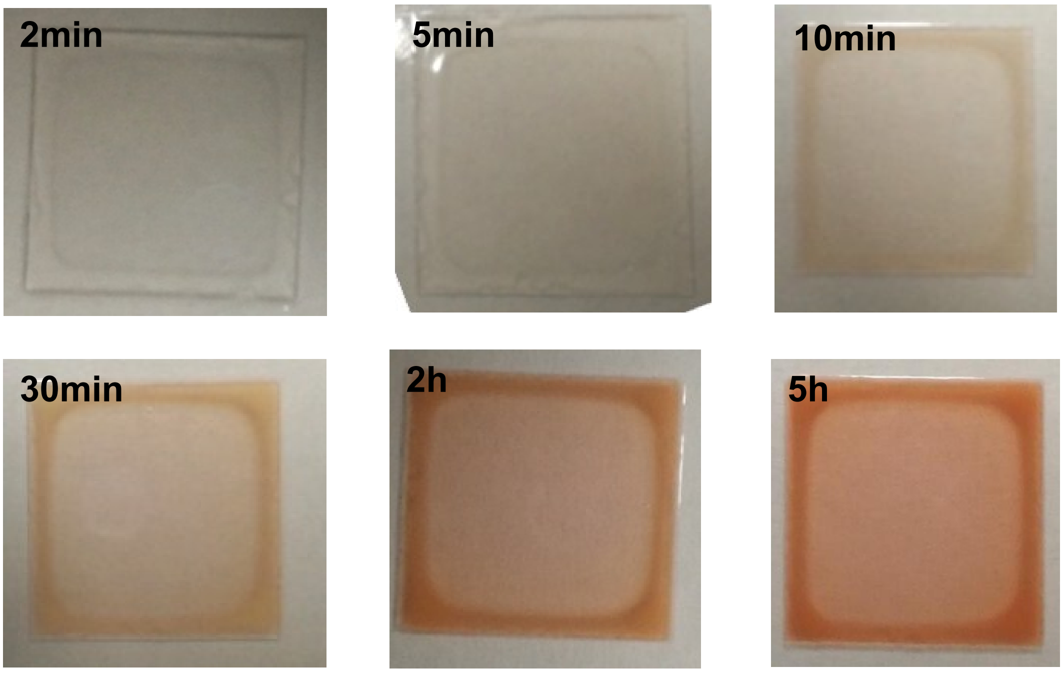

Figure S1. The photograph of the ethanolic solution containing resol, P123 and $ZnCl_2$ which was drop-cast on the surface of a glass slide with a size of 2 cm × 2 cm and subjected to themopolymerization at 100 °C for different durations. After ~ 2 min, the solution was turned to be a transparent, sticky film. After ~ 5 min, the film took a slightly brownish color and was no longer sticky. The film exhibited increasingly darker color until thermopolymerization for 5 h. This observation indicates that thermopolymerization of resol starts almost instantly upon heating at 100 °C.

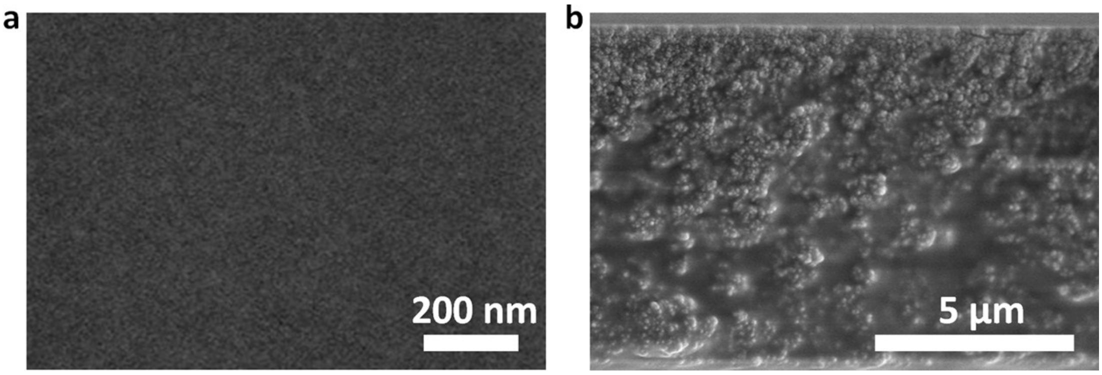

Figure S2. The SEM images of (a) the surface and (b) the cross section of the as-synthesized phenolic film before acid soaking.

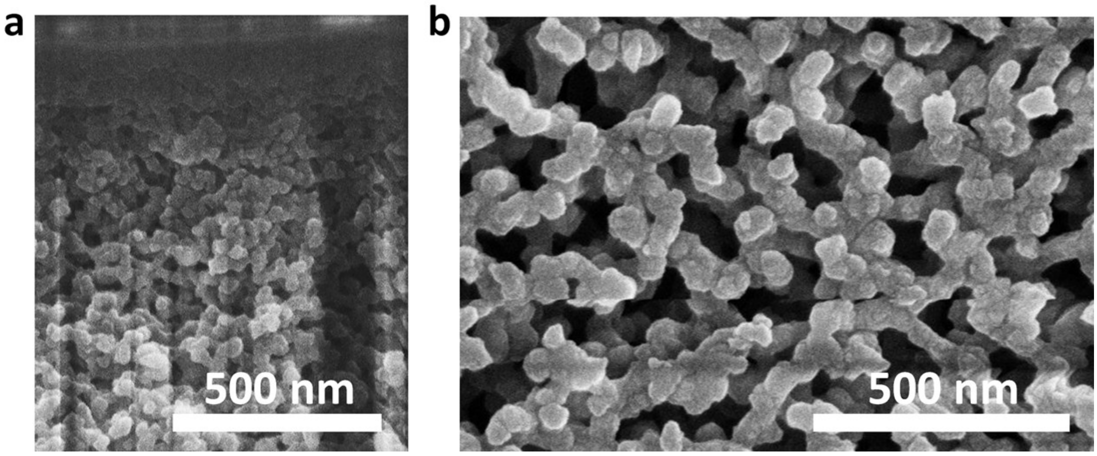

Figure S3. The SEM images of (a) the cross section and (b) the bottom surface of the phenolic membrane prepared by soaking in water for 2 h at room temperature.

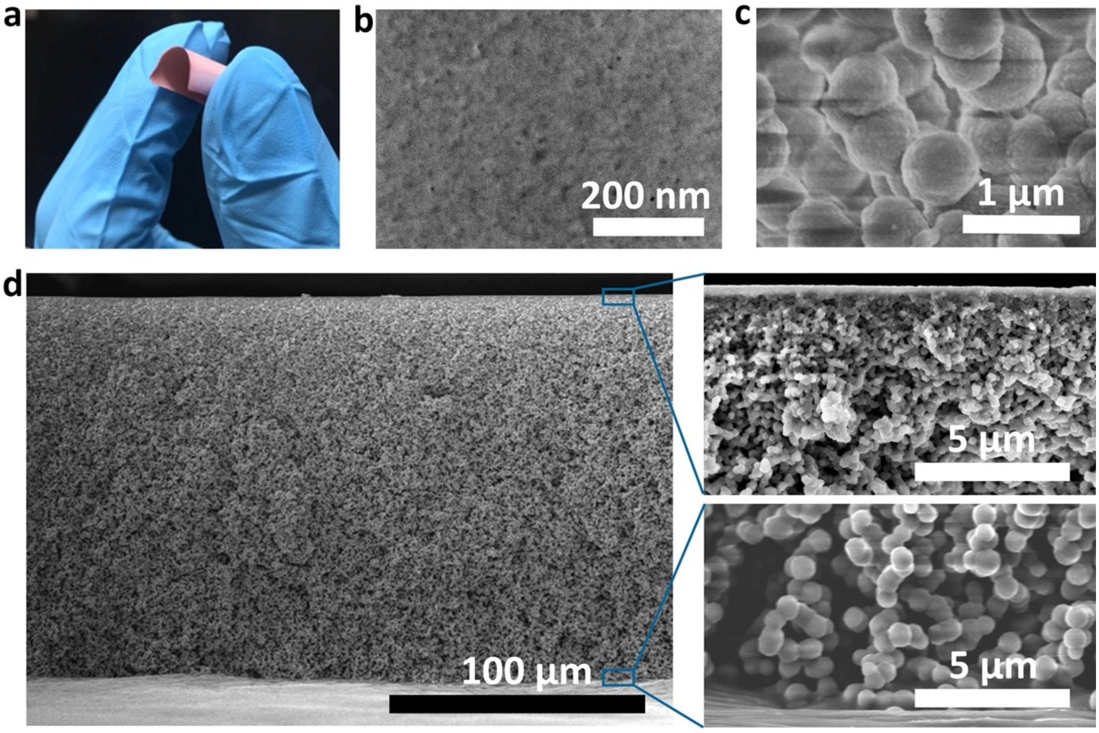

Figure S4. The bulk phenolic membrane with a thickness of ~ 150 µm prepared by casting 400 µL resol solution on a 2 cm × 2 cm substrate, followed by thermopolymerization and H_2SO_4 soaking. The photograph (a); The SEM image of the (b) top and (c) bottom surface; The SEM images of the cross section (d).

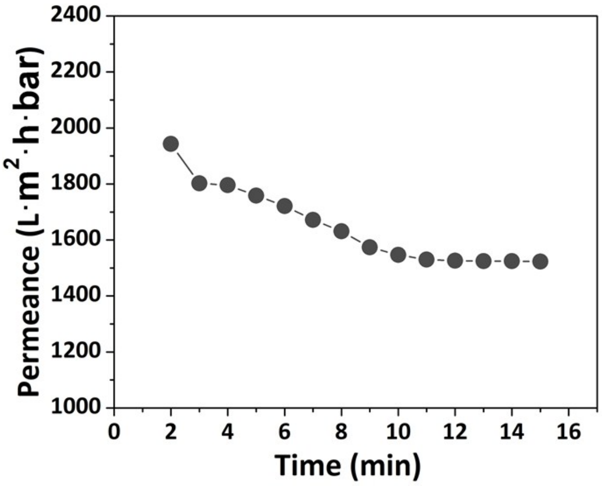

Figure S5. Time variation of permeance of the phenolic membrane prepared by soaking in 55% H_2SO_4 at 100 ºC for 2 h under the pressure of 0.4 bar.

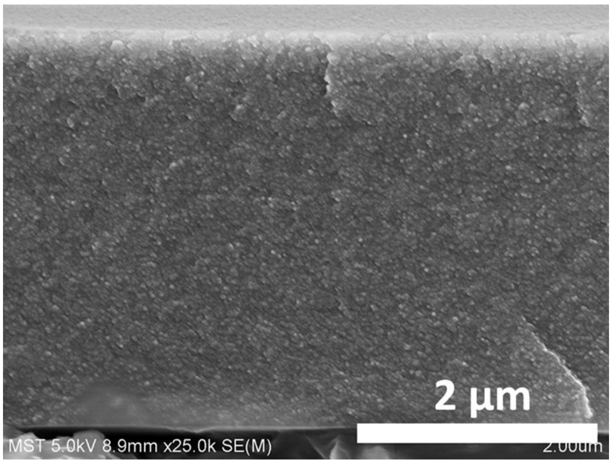

Figure S6. The cross-sectional SEM image of the phenolic film prepared by slow evaporation of ethanol at 20 °C followed by thermopolymerization and H_2SO_4 soaking.

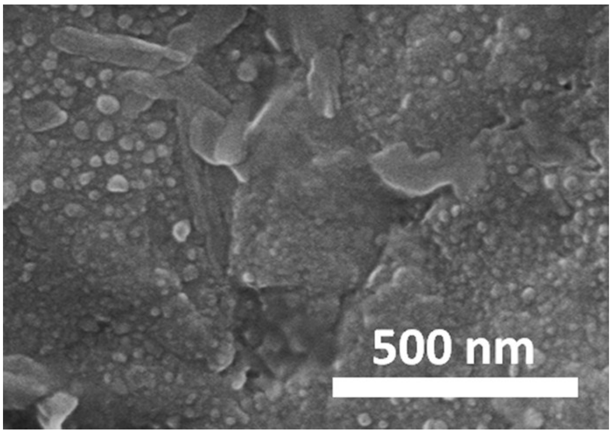

Figure S7. The cross-sectional SEM image of the phenolic film prepared without $ZnCl_2$. The film has been soaked in 55% H_2SO_4 at 100 ºC for 2 h.

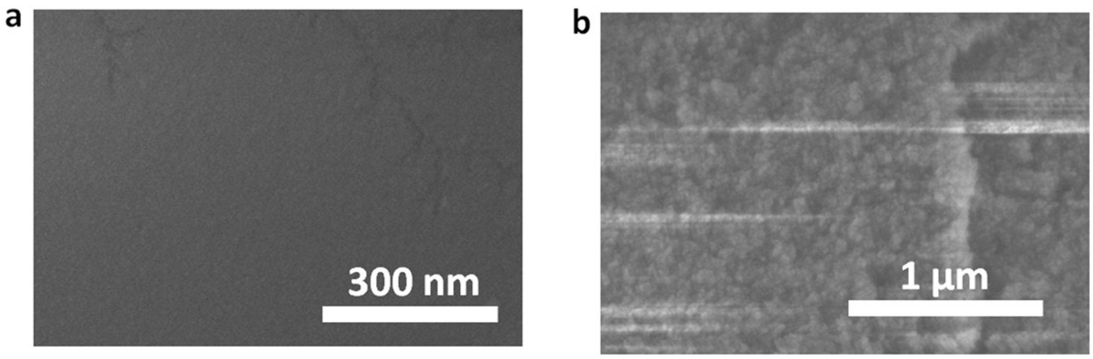

Figure S8. The SEM images of the (a) bottom surface and (b) cross section of phenolic film prepared without P123. The film has been soaked in 55% H_2SO_4 at 100 ºC for 2 h.

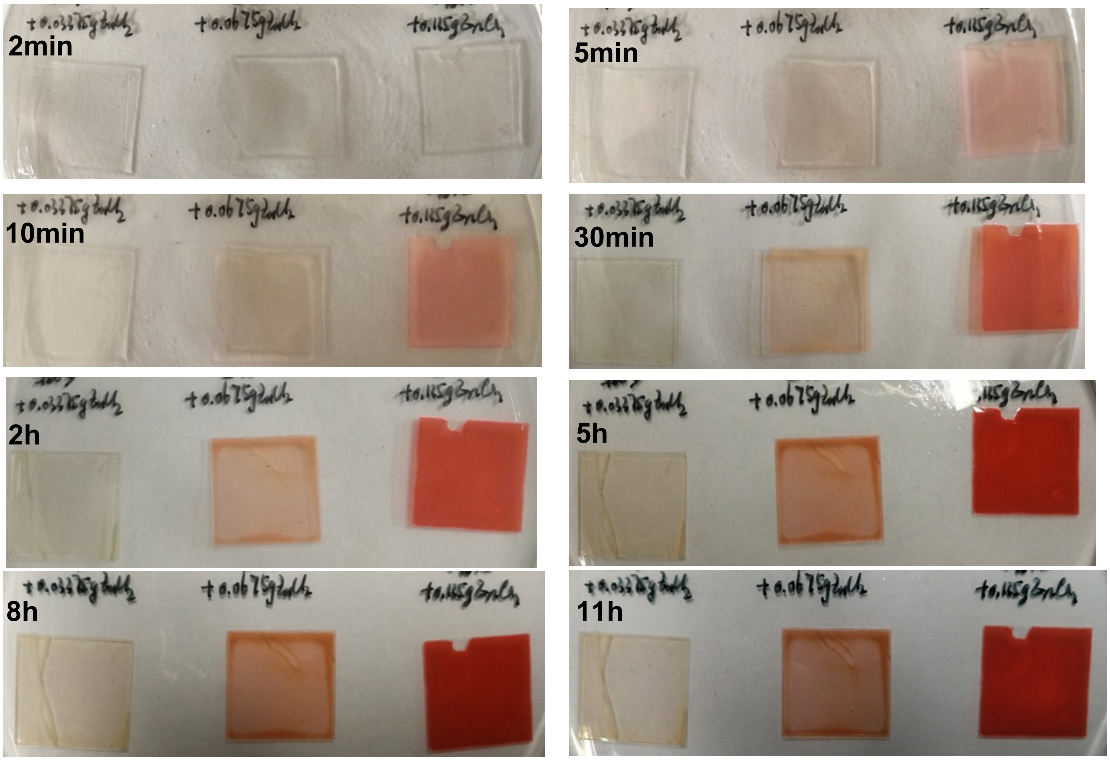

Figure S9. The photographs of the ethanolic solutions of resol with various molar ratios of $ZnCl_2$ to P123 which was drop-cast on the surface of a glass slide and subjected to themopolymerization at 100 °C for different durations. For each panel, from left to right, the samples were prepared at the molar ratio of 70, 140, and 280, respectively.

S10. Estimation of the porosity of the phenolic membranes prepared with different molar ratios of $ZnCl_2$ to P123.

Porosity in this work means the ratio of the volume occupied by all the pores in the phenolic membrane to the total volume of the membrane. Therefore, it is an averaged parameter indicating the porous nature of the phenolic membrane, collectively reflecting the porous nature of the entire membrane. Therefore, it does not describe the change of pore size across the thickness of a specific membrane sample. Instead, porosity is used to indicate the difference in the degree of the porous state of different membrane samples prepared with changing $ZnCl_2$ dosages, thus revealing the effect of $ZnCl_2$ dosages to the porous structure of the phenolic membranes.

As the generation of the pores in the phenolic membranes is due to the introduction of $ZnCl_2$, we can easily estimate the porosity by comparing the thicknesses of the membranes prepared with and without $ZnCl_2$.

$$\delta=(t_1-t_2)/t_1 \quad \text{(S2)}$$

where δ is the porosity of the phenolic membranes. t_1 represents the thickness of the phenolic membranes prepared with the molar ratios of $ZnCl_2$ to P123 from 70 to 280, while t_2 is the thickness of the membranes prepared without $ZnCl_2$. The calculated results are shown as below:

Molar ratios of $ZnCl_2$ to P123	Thickness (*t, μm*)	Porosity (*δ, %*)
0	2.9	0
70	4.7	38.3
140	10	71
280	12	75.8

The thicknesses were measured by SEM imaging and the results showed very little variation. Consequently, the porosity estimated from the value of thickness was stable. Therefore, we did not include error bars into Fig. 6a.

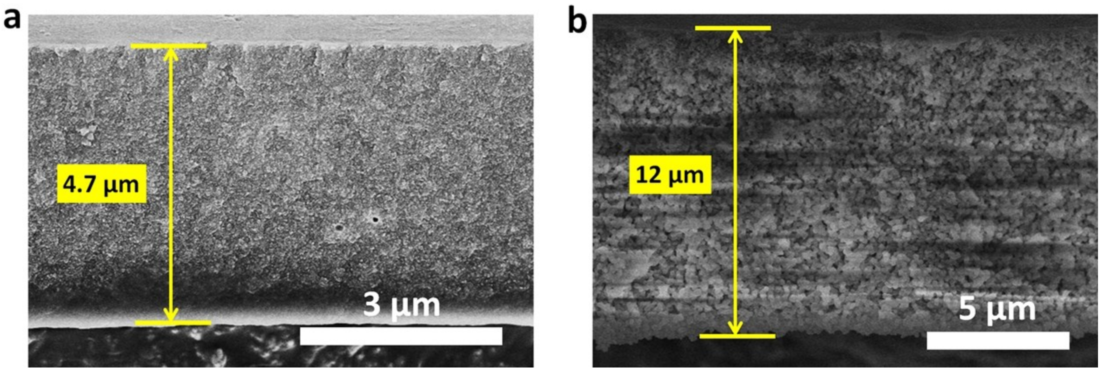

Figure S11. The cross-sectional SEM images of phenolic membranes prepared with different molar ratios of $ZnCl_2$ to P123: (a) molar ratio=70; (b) molar ratio=280. The membrane has been soaked in 55% H_2SO_4 at 100 ºC for 2 h.

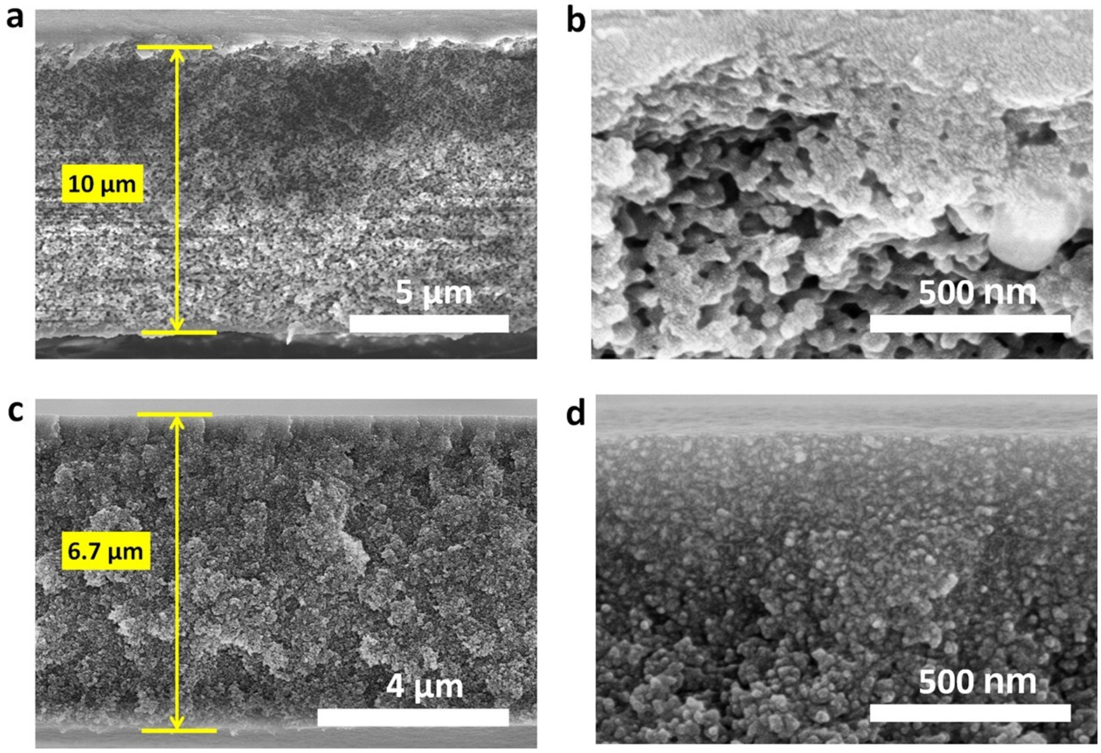

Figure S12. The cross-sectional SEM images of phenolic membranes prepared with various molar ratios of P123 to phenol: (a, b) molar ratio = 0.012; (c, d) molar ratio = 0.024. The membrane has been soaked in 55% H_2SO_4 at 100 ºC for 2 h.

Table S1. Comparison of water permeances and BSA or Cyt.c rejections of gradient nanoporous phenolic membrane prepared in this work and other membranes.

Membrane	Water permeance (L/(bar·m^2·h))	Rejection (%)	Rejection solute	Reference
PVC/PVF	323.6	90.2	BSA	6
PVDF/GO@SiO$_2$/PVP	1232	77.5	BSA	7
SWCNT-PANi	1367.5	28	BSA	8
PBI blended PSf	177.5	68.7	BSA	9
Isoporous PS-*b*-PEO	800	67	BSA	10
Polyzwitterion grafted PEI	264	99	BSA	11
PPSU	171	94	BSA	12
PAN-AA-Mg	70.7	97.8	BSA	13
PVDF-MWCNTs	620	89	BSA	14
PVDF−GO	457.9	91.1	BSA	15
GO	398.7	92.6	BSA	16
BCP micelle	710	30	Cyt.c	17
MXene	1056	97	Cyt.c	18
SPEK-C	534.6	82	Cyt.c	19
NSC-GO	568	98	Cyt.c	20
SWCNT-intercalated GO	700	98.3	Cyt.c	21
Layed WS$_2$ nanosheet	725	91	Cyt.c	22
Laminar MoS$_2$	245	98	Cyt.c	23
C$_3$N$_4$	29	93.1	Cyt.c	24
Gradient nanoporous phenolic	1547	96<br>95	BSA<br>Cyt.c	This work

Reference

1 Y. Meng, D. Gu, F. Zhang, Y. Shi, H. Yang, Z. Li, C. Yu, B. Tu and D. Zhao, Ordered Mesoporous Polymers and Homologous Carbon Frameworks: Amphiphilic Surfactant Templating and Direct Transformation, *Angew. Chem. Int. Ed.*, 2005, **117**, 7053-7059.

2 S. Plimpton, Fast Parallel Algorithms for Short-Range Molecular Dynamics, *J. Computat. Phys.*, 1995, **117**, 1-19.

3 H. Sun, Force Field for Computation of Conformational Energies, Structures, and Vibrational Frequencies of Aromatic Polyesters, *J. Comput. Chem.*, 1994, **15**, 752-768.

4 H. Sun, S. J. Mumby, J. R. Maple and A. T. Hagler, An ab Initio CFF93 All-Atom Force Field for Polycarbonates, *J. Am. Chem. Soc.*, 1994, **116**, 2978-2987.

5 H. Sun, S. J. Mumby, J. R. Maple and A. T. Hagler, Ab Initio Calculations on Small Molecule Analogs of Polycarbonates, *J. Phys. Chem.*, 1995, **99**, 5873-5882.

6 X. Fan, Y. Su, X. Zhao, Y. Li, R. Zhang, J. Zhao, Z. Jiang, J. Zhu, Y. Ma and Y. Liu, Fabrication of Polyvinyl Chloride Ultrafiltration Membranes with Stable Antifouling Property by Exploring the Pore Formation and Surface Modification Capabilities of Polyvinyl Formal, *J. Membr. Sci.*, 2014, **464**, 100-109.

7 Z. Zhu, J. Jiang, X. Wang, X. Huo, Y. Xu, Q. Li and L. Wang, Improving the

Hydrophilic and Antifouling Properties of Polyvinylidene Fluoride Membrane by Incorporation of Novel Nanohybrid GO@SiO_2 Particles, *Chem. Eng. J.*, 2017, **314**, 266-276.

8 Y. Liao, D. Yu, X. Wang, W. Chain, X. Li, E. M. Hoek and R. B. Kaner, Carbon Nanotube-Templated Polyaniline Nanofibers: Synthesis, Flash Welding and Ultrafiltration Membranes, *Nanoscale*, 2013, **5**, 3856-3862.

9 E. Eren, A. Sarihan, B. Eren, H. Gumus and F. O. Kocak, Preparation, Characterization and Performance Enhancement of Polysulfone Ultrafiltration Membrane Using PBI as Hydrophilic Modifier, *J. Membr. Sci.*, 2015, **475**, 1-8.

10 M. Karunakaran, S. P. Nunes, X. Qiu, H. Yu and K.-V. Peinemann, Isoporous PS-*b*-PEO Ultrafiltration Membranes via Self-Assembly and Water-Induced Phase Separation, *J. Membr. Sci.*, 2014, **453**, 471-477.

11 N. L. Le, M. Ulbricht and S. P. Nunes, How Do Polyethylene Glycol and Poly(Sulfobetaine) Hydrogel Layers on Ultrafiltration Membranes Minimize Fouling and Stay Stable in Cleaning Chemicals? *Ind. Eng. Chem. Res.*, 2017, **56**, 6785-6795.

12 A. K. Shukla, J. Alam, M. Alhoshan, L. A. Dass and M. R. Muthumareeswaran, Development of A Nanocomposite Ultrafiltration Membrane Based on Polyphenylsulfone Blended with Graphene Oxide, *Sci. Rep.*, 2017, **7**, 41976-41987.

13 Y. Yang, X. Li, L. Shen, X. Wang and B. S. Hsiao, Ionic Cross-Linked

Poly(Acrylonitrile-Co-Acrylic Acid)/Polyacrylonitrile Thin Film Nanofibrous Composite Membrane with High Ultrafiltration Performance, *Ind. Eng. Chem. Res.*, 2017, **56**, 3077-3090.

14 Y. Zhao, Z. Xu, M. Shan, C. Min, B. Zhou, Y. Li, B. Li, L. Liu and X. Qian, Effect of Graphite Oxide and Multi-Walled Carbon Nanotubes on the Microstructure and Performance of PVDF Membranes, *Sep. Purif. Technol.*, 2013, **103**, 78-83.

15 Z. Wang, H. Yu, J. Xia, F. Zhang, F. Li, Y. Xia and Y. Li, Novel GO-Blended PVDF Ultrafiltration Membranes, *Desalination*, 2012, **299**, 50-54.

16 Z. Xu, T. Wu, J. Shi, W. Wang, K. Teng, X. Qian, M. Shan, H. Deng, X. Tian, C. Li and F. Li, Manipulating Migration Behavior of Magnetic Graphene Oxide via Magnetic Field Induced Casting and Phase Separation toward High-Performance Hybrid Ultrafiltration Membranes, *ACS Appl. Mater. Interfaces*, 2016, **8**, 18418-18429.

17 X. Yao, L. Guo, X. Chen, J. Huang, M. Steinhart and Y. Wang, Filtration-Based Synthesis of Micelle-Derived Composite Membranes for High-Flux Ultrafiltration, *ACS Appl. Mater. Interfaces*, 2015, **7**, 6974-6981.

18 L. Ding, Y. Wei, Y. Wang, H. Chen, J. Caro and H. Wang, Two-Dimensional Lamellar Membrane: Mxene Nanosheet Stacks., *Angew. Chem. Int. Ed.*, 2017, **56**, 1825-1829.

19 C. Deng, Q. Zhang, G. Han, Y. Gong, A. Zhu and Q. Liu, Ultrathin Self-Assembled Anionic Polymer Membranes for Superfast Size-Selective

Separation, *Nanoscale*, 2013, **5**, 11028-11034.

20 H. Huang, Z. Song, N. Wei, L. Shi, Y. Mao, Y. Ying, L. Sun, Z. Xu and X. Peng, Ultrafast Viscous Water Flow Through Nanostrand-Channelled Graphene Oxide Membranes., *Nat. Commun.*, 2013, **4**, 2979-2988.

21 S. Gao, H. Qin, P. Liu and J. Jin, SWCNT-Intercalated GO Ultrathin Films for Ultrafast Separation Of Molecules, *J. Mater. Chem. A*, 2015, **3**, 6649-6654.

22 L. Sun, Y. Ying, H. Huang, Z. Song, Y. Mao, Z. Xu and X. Peng, Ultrafast Molecule Separation Through Layered WS_2 Nanosheet Membranes, *ACS Nano*, 2014, **8**, 6304-6311.

23 L. Sun, H. Huang and X. Peng, Laminar Mos_2 Membranes for Molecule Separation, *Chem. Commun.*, 2013, **49**, 10718-10720.

24 Y. Wang, L. Li, Y. Wei, J. Xue, H. Chen, L. Ding, J. Caro and H. Wang, Water Transport with Ultralow Friction Through Partially Exfoliated G-C_3N_4 Nanosheet Membranes with Self-Supporting Spacers, *Angew. Chem. Int. Ed.*, 2017, **56**, 8974-8980.
